# Supplementary material for: Patterns of Intron Gain and Loss in Fungi
Source: PLoS Biol. 2004 Nov 30;2(12):e422. doi: 10.1371/journal.pbio.0020422 (PMC532390; doi:10.1371/journal.pbio.0020422)
Supplement: Table S1 — Also available at http://genes.mit.edu/NielsenEtAl/. (4.3 MB ZIP). [file pbio.0020422.st001.zip › NielsenEtAl/html/202.html]

AN3756.1.NCU09290.1.MG02086.1.FG05631.1


```
 CLUSTAL W (1.82) Multiple Sequence Alignments - Introns Inserted


Sequence 1: MG02086.1	211 aa
Sequence 2: FG05631.1	212 aa
Sequence 3: NCU09290.1	257 aa
Sequence 4: AN3756.1	233 aa
Alignment Length: 259 aa
Number Identitical Residues: 158 aa
Alignment Score (without introns) 6702


MG02086.1 	--------------~-----MDRLKKGEVN2LGTSI2MAVTFKDGVILG1ADSRTTTGAY
NCU09290.1	--MEFGTSGVLSED1GIHVDMDRLKKGEVN2LGTSI2MAVTFKDGVILG1ADSRTTTGAY
FG05631.1 	--------------~-----MDRLKKGEVN2LGTSI2MAVTFKDGVILG1ADSRTTTGAY
AN3756.1  	MVASHGMTGMLGED1GIHIDMNHLKSGEVN2LGTSI2MAINFKDGVILG~ADSRTTTGAY
          	   . . :.  ... .   .*::**.**** ***** **:.******** **********

MG02086.1 	IANRVTDKLTRVSDTIWCCRSGSAADTQAVADIVQYQLGLFHMTNGKPPTTQTAAAIFQE
NCU09290.1	IANRVTDKLTQVHDTIWCCRSGSAADTQAVADIVQYQLGLYHMVNGRPPTTQIAAAVFQE
FG05631.1 	IANRVTDKLTRVHDTIWCCRSGSAADTQAVADIVQYQLGLFAMTNGKPPMTQTAASIFQE
AN3756.1  	IANRVTDKLTQVHDTIWCCRSGSAADTQAVADIVSYHLNMYSIVNNEPPSTQVAAALFQE
          	**********:* *********************.*:*.:: :.*..** ** **::***

MG02086.1 	ICYSNKDRLS2AGLIIAGWDERHGGQVYSIPLGGSLHKQAYAIGGSGSTYIYGYCDANWR
NCU09290.1	IAYANKDRLT2AGLIIAGWDARHGGQVYNIPLGGSLHKQPYSIGGSGSTYIYGYCDANWK
FG05631.1 	ICYANKDRLS2AGLIIAGWDERFGGQVYSIPLGGSLHKQSYAIGGSGSTYIYGYCDANWR
AN3756.1  	LCYENKDMLS2AGIIIAGYDPRHGGQVYSIPLGGSLHKQAYSIGGSGSTYIYGYCDANWK
          	:.* *** *: **:****:* *.*****.**********.*:*****************:

MG02086.1 	EGMEEADAVQFVKGALAEAIKWDGSSGGVIRMVVLTAKGADRHLYLPDEGY-~------T
NCU09290.1	EGMEEAEAVAFVKEALKEAIKWDGSSGGVIRMVVLTEKGADRHLYLPDSDYK0LEVVPHI
FG05631.1 	EGMEKDDAVNFVKGALREAIKWDGSSGGVIRMVVLTKEGADRHLYLPDTDY-~------A
AN3756.1  	ENMTEEEGVEFVRGALREAIKWDGSSGGVIRMVVLTKNGAQRHLYLPDNGY-~------T
          	*.* : :.* **: ** ******************* :**:******* .*         

MG02086.1 	VKH----------~------------
NCU09290.1	LEHEDKFAFIHIH1EAWRTRREVGDG
FG05631.1 	VRHE---------~------------
AN3756.1  	APGRTN-------~------------
          	     .
```
